# Innovative therapeutic strategies for cardiovascular disease

**DOI:** 10.17179/excli2023-6306

**Published:** 2023-07-26

**Authors:** Kenneth Maiese

**Affiliations:** 1 Cellular and Molecular Signaling, New York, New York 10022

**Keywords:** AMPK, apoptosis, autophagy, FoxO, NAD+, SIRT1

## Abstract

As a significant non-communicable disease, cardiovascular disease is the leading cause of death for both men and women, comprises almost twenty percent of deaths in most racial and ethnic groups, can affect greater than twenty-five million individuals worldwide over the age of twenty, and impacts global economies with far-reaching financial challenges. Multiple factors can affect the onset of cardiovascular disease that include high serum cholesterol levels, elevated blood pressure, tobacco consumption and secondhand smoke exposure, poor nutrition, physical inactivity, obesity, and concurrent diabetes mellitus. Yet, addressing any of these factors cannot completely eliminate the onset or progression of cardiovascular disorders. Novel strategies are necessary to target underlying cardiovascular disease mechanisms. The silent mating type information regulation 2 homolog 1* (Saccharomyces cerevisiae*) (SIRT1), a histone deacetylase, can limit cardiovascular injury, assist with stem cell development, oversee metabolic homeostasis through nicotinamide adenine dinucleotide (NAD^+^) pathways, foster trophic factor protection, and control cell senescence through the modulation of telomere function. Intimately tied to SIRT1 pathways are mammalian forkhead transcription factors (FoxOs) which can modulate cardiac disease to reduce oxidative stress, repair microcirculation disturbances, and reduce atherogenesis through pathways of autophagy, apoptosis, and ferroptosis. AMP activated protein kinase (AMPK) also is critical among these pathways for the oversight of cardiac cellular metabolism, insulin sensitivity, mitochondrial function, inflammation, and the susceptibility to viral infections such as severe acute respiratory syndrome coronavirus that can impact cardiovascular disease. Yet, the relationship among these pathways is both intricate and complex and requires detailed insight to successfully translate these pathways into clinical care for cardiovascular disorders.

## The Global Implications for Cardiovascular Disease

Non-communicable diseases (NCDs) affect an increasing portion of the global population with at least fifteen million of these individuals ranging in age between thirty and sixty-nine years (Jalgaonkar et al., 2022[110]; Maiese, 2018[193], 2020[165], 2021[197]; Schell et al., 2021[276]; Speer et al., 2020[294]). In developed nations, NCDs affect at least ten percent of individuals that are less than sixty years of age. However, in low and middle-income countries, NCDs affect a greater proportion of people with at least one-third of the population affected under sixty years of age (WHO, 2011[315], 2017[314]). 

In particular, cardiovascular disorders have a significant role in NCDs. NCDs are a primary cause of death that include cardiac disease, cancer, trauma, respiratory disease, stroke, Alzheimer's disease, diabetes mellitus (DM), influenza and pneumonia, kidney disease, and suicide (CDC, 2019[30] (Table 1(Tab. 1))). Cardiovascular disease is the leading cause of death for both women and men and accounts for more than forty percent of all deaths when combined with the deaths from cancer (Chen et al., 2023[36]; Huang et al., 2023[106]; Kalam et al., 2023[117]; Kostić et al., 2023[124]; Liu et al., 2023[153]; Maiese, 2015[166][191], 2017[171]; Ponzetti et al., 2023[251]; Razzaghi et al., 2023[260]; Redhwan et al., 2023[261]; Sierra-Pagan et al., 2023[290]; Yeger, 2023[328]; Zhong et al., 2023[348]). It is estimated that an individual dies every thirty-three seconds as a result of heart disease in the United States (US) alone (Heron, 2019[98]; CDC, 2023[29]). In 2021, approximately 700,000 individuals expired from cardiac disease. More than eighteen million individuals over the age of twenty experience coronary artery disease in the US and greater than 800,000 people have a myocardial infarction every year. Although ethnicity can play a role in the percentage of deaths, cardiovascular disease affects most racial and ethnic groups in at least 10 % of these populations with African Americans being affected to the highest degree at almost 23 %. In regard to financial care concerns, healthcare costs for cardiovascular disease exceed $ 555 billion US dollars and by the year 2035 they will be greater than $ 1.1 trillion US dollars.

## Cardiovascular Disease and the Aging Process

Closely tied to the increasing prevalence of NCDs and cardiovascular disease are the effects of the aging process (Amidfar et al., 2023[8]; Blice-Baum et al., 2017[20]; Maiese, 2015[191], 2023[158]; Olejniczak et al., 2023[238]; Rotllan et al., 2021[266]; Sun et al., 2023[296]; Wang et al., 2021[308]). On one side of the equation for the aging process, lifespan is increasing throughout the world with the expectation of attaining at least 80 years of age (Geier and Perl, 2021[79]; Gustafsson and Ulfhake, 2021[90]; Jalgaonkar et al., 2022[110]; Maiese, 2014[162], 2021[197]; Yu et al., 2021[330]). Even in developing countries such as India and China, the elderly population is expected to increase from five to ten percent over future years (Maiese, 2015[191], 2017[175]). Across the globe, the number of individuals over the age of 65 has doubled during the previous 50 years (Hayutin, 2007[95]). Multiple factors have contributed to increased life expectancy with cardiovascular disease, except for the growing non-prescribed use of fentanyl by individuals that has resulted in a rise in mortality in this group (Wilson et al., 2020[316]). Factors that have positively promoted increased lifespan include incorporation of early diagnostic and preventive measures, rapidly identifying individuals susceptible to both acute and chronic illnesses, improved sanitation, and improved access to healthcare (Chen et al., 2023[36]; Hacioglu et al., 2021[91]; Jalgaonkar et al., 2022[110]; Jiang et al., 2023[114]; Kahmini et al., 2022[116]; Li et al., 2023[136]; Liu et al., 2022[144]; Maiese, 2018[200], 2021[160][177], 2022[205]; Odnokoz et al., 2021[234]; Patocka et al., 2021[246]; Sorrells et al., 2021[293]). 

**Figure 1:**
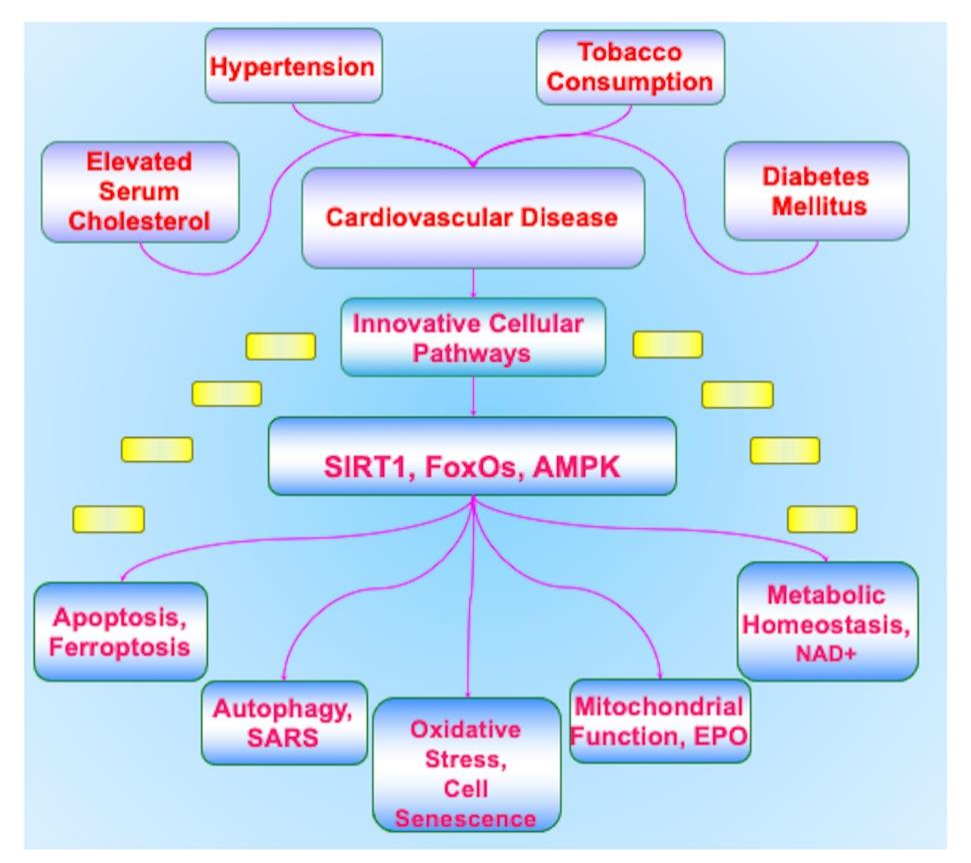
Novel therapeutic strategies for cardiovascular disease Cardiovascular disease is impacted by multiple risk factors that include elevated serum cholesterol, hypertension, tobacco consumption, and concurrent disorders such as diabetes mellitus that treatment of these cannot completely avert the development of cardiovascular disease. Innovative cellular pathways that involve the silent mating type information regulation 2 homolog 1* (Saccharomyces cerevisiae*) (SIRT1), mammalian forkhead transcription factors (FoxOs), and AMP activated protein kinase (AMPK) are required to address the underlying critical mechanisms for cardiovascular disease. SIRT1, FoxOs, and AMPK can oversee vital critical pathways for cardiovascular survival that involve nicotinamide adenine dinucleotide (NAD^+^), oxidative stress, cell senescence, mitochondrial function, trophic factor protection such as with erythropoietin (EPO), infectious agent injury such as with severe acute respiratory syndrome coronavirus (SARS-CoV-2), and the programmed cell death pathways that include apoptosis, autophagy, and ferroptosis.

Yet, on the other side of the equation that involves aging are the underlying cellular pathways that oversee an individual's longevity (Figure 1(Fig. 1)). These cellular pathways can affect the onset and progression of cardiac disease (Blice-Baum et al., 2017[20]; Choudhery et al., 2012[51]; Du et al., 2016[64]; Maiese, 2016[185]; Okada et al., 2016[237]). Recent work has focused on the role of telomeres (TLs), complexes of deoxyribonucleic acid (DNA), that can oversee cell senescence, control stem cell renewal, cellular lifespan, cellular replication, and protection for the DNA of the genome (Alanko et al., 1996[4]; Bandara and La Thangue, 1991[12]; Chen et al., 1997[34]; Connor et al., 2001[53]; Dhakal et al., 2019[58]; Ferrara-Romeo et al., 2020[72]; Jaganjac et al., 2022[108]; Jeyaraman et al., 2022[112]; Khani et al., 2022[121]; Kita et al., 2022[122]; Li et al., 2021[137]; Mahdi et al., 1995[155]; Maiese, 2020[182][189], 2023[199]; Maiese et al., 2010[208]; O'Donnell et al., 2022[235]; Okada et al., 2016[237]; Oyefeso et al., 2021[240]; Puri et al., 2023[253]; Saxton and Pawson, 1999[275]; Sun et al., 2023[296]; Takanezawa et al., 2021[299]; Topiwala et al., 2023[304]; Wang et al., 2023[306]; Yan et al., 2021[323]; Zhao et al., 2013[345]). In patients with dilated cardiomyopathy, decreased TL length and progression of cell senescence has been observed (Barcena et al., 2023[13]) (Table 1(Tab. 1)). TLs exist at the end of chromosomes, have greater than 2000 repetitions of non-coding double-stranded DNA with the sequence 'TTAGGG”, and are completed with a guanine rich single-stranded DNA (Dhakal et al., 2019[58]; Kuan et al., 2023[126]). A number of protein complexes are associated with TLs that include telosome, shelterin, and CTC1-STN1-TEN1 (CST). These proteins control TLs activity and provide stability. The telomerase protein becomes active during cell division to maintain TL length through the addition of tandem repeat ribonucleic acid (RNA) templates. Without this process, a portion of TLs length will become lost in the amount of approximately 25-200 base pairs (Cardoso et al., 2021[26]; De Bonis et al., 2014[57]; Klionsky et al., 2021[123]; Shafi, 2016[280]). If telomerase function is also lost or the TLs become excessively short with less than 500 base pairs, cell proliferation is no longer viable and cell senescence ensues (Begum et al., 2021[16]; Cai,et al., 2021[23]; Dorvash et al., 2020[61]; Geng et al., 2021[80]; Kowalska et al., 2020[125]; Liu et al., 2020[149]; Maiese, 2014[164], 2015[194], 2016[196], 2020[189]; Rapaka et al., 2022[258]; Tabibzadeh, 2021[298]; Yu et al., 2021[330]; Zhang et al., 2020[337]; Zhou et al., 2022[349]). With the onset of cell senescence, cardiac injury can develop since reparative processes are unable to function (Blice-Baum et al., 2017[20]; Du et al., 2016[64]; Lathe and St Clair, 2023[130]; Maiese, 2014[164], 2015[170], 2016[163][198][185][196], Maiese et al., 2008[207]; Okada et al., 2016[237]; Sun et al., 2023[296]; Yamamoto et al., 2023[321]). In addition, exposure to oxidative stress can develop with the release of reactive oxygen species (ROS) during the shortening of TLs and the onset of cell senescence. Oxidative stress exposure leads to decreased cell survival and the dysfunction of mitochondrial organelles (Cardoso et al., 2021[26]; Chen et al., 2022[37]; Fields et al., 2019[75]; Gallyas et al., 2020[78]; Groen et al., 2022[86]; Lei et al., 2021[133]; Li et al., 2020[138][140]; Liu et al., 2020[149]; Maiese, 2016[198], 2020[201][189], 2021[181]; Mocayar Marón et al., 2020[226]; Odnokoz et al., 2021[234]; Oliveira et al., 2021[239]; Oyefeso et al., 2021[240]; Perluigi et al., 2021[248]; Piao et al., 2021[249]; Prasuhn and Brüggemann, 2021[252]; Raut and Khullar, 2023[259]; Tabibzadeh, 2021[298]; Xiong et al., 2022[317]; Zhang et al., 2020[337]; Zhuang et al., 2022[351]) (Figure 1(Fig. 1)). The inability to remove cells that are senescent by the immune system also may subsequently lead to tumorigenesis (Begum et al., 2021[16]; Cai et al., 2021[23]; Kowalska et al., 2020[125]; Liu et al., 2020[149]; Maiese, 2016[163][198][185], 2020[189]; Watroba and Szukiewicz, 2021[312]; Yu et al., 2021[330]; Zhang et al., 2020[337]). 

## Innovative Therapeutic Strategies for Cardiovascular Disease

Multiple factors can lead to the onset of cardiovascular disease. Focusing upon adults who are male or female, age sixty and over, or non-Hispanic black, or of lower income who are at high risk may reduce the onset and progression of cardiovascular disease (Fryar et al., 2012[76]; Maiese, 2008[203], 2020[178]). New imaging technology involving multiphoton microscopy and optogenetic effectors and sensors can offer improved observation of cardiac dynamics and cellular signaling (Lee et al., 2021[131]). Pulsed lasers offer the ability to possibly treat atherosclerotic plaques (Sintek et al., 2021[291]). Additional therapeutic pathways can reduce the risk of cardiovascular disorders by addressing serum cholesterol levels, elevated blood pressure, tobacco consumption and secondhand smoke exposure, poor nutrition, physical inactivity, obesity, and the presence of DM (Ahmed et al., 2020[3]; Begum et al., 2021[16]; Chong and Maiese, 2012[48]; Chong et al., 2011[49]; du Toit et al., 2022[63], 2023[62]; Januszewski et al., 2020[111]; Liu et al., 2020[147]; Maiese, 2016[185][190], 2018[200], 2019[173][174]; Maiese et al., 2008[207], 2009[209][218]; Najjar et al., 2021[230]; Quintana-Pérez et al., 2022[255]; Ran et al., 2021[257]; Raut and Khullar, 2023[259]; Rotllan et al., 2021[266]; Su et al., 2022[295]; Temiz- Resitoglu et al., 2022[302]; Wang et al., 2021[308]; Zhang et al., 2023[338]) (Figure 1(Fig. 1)). Treatment of metabolic disorders that involves DM represents a critical pathway to reduce cardiovascular disease since DM leads to the progression of cardiovascular injury. In fact, individuals with DM are twice as likely to suffer from cardiac disease or stroke when compared to individuals without DM (Hajibabaie et al., 2022[92]; Maiese, 2015[176], Maiese et al., 2009[209][214]; Pabel et al., 2021[241]; Rotllan et al., 2021[266]; Xue et al., 2019[320]). The implementation of pharmaceuticals and nutritional modification can assist with the management of DM to prevent hyperglycemic events (Arildsen et al., 2019[10]; Bayaraa et al., 2022[14]; Beegum et al., 2022[15]; Chen et al., 2021[35]; Chiareli et al., 2021[38]; Esterline et al., 2018[67]; Feng et al., 2020[71]; Gong et al., 2021[84]; Hajibabaie et al., 2022[92]; Jalgaonkar et al., 2022[110]; Jiang et al., 2023[114]; Liu et al., 2020[148]; Maiese, 2018[193], 2020[189], 2021[181]; Maiese et al., 2008[212][215]; Mocayar Marón et al., 2020[226]; Pabel et al., 2021[241]; Papachristoforou et al., 2020[244]; Rotllan et al., 2021[266]; Sakakibara et al., 2002[268]; Sanabria-de la Torre et al., 2022[272]; Tan et al., 2021[300]; Zaiou, 2020[333]; Zarneshan et al., 2020[334]; Zhou et al., 2021[350]). However, even therapies designed to effectively manage poor glycemic control are not without risks. These therapeutic regimens can affect cellular organelles and lead to decreased organ mass through processes that involve programmed cell death and autophagy (Gong et al., 2021[84]; Lee et al., 2014[132]; Li et al., 2020[135]; Mocayar Marón et al., 2020[226]) (Figure 1(Fig. 1)). With these challenges at hand, new and innovative strategies are highly warranted that can address the underlying cellular components of cardiovascular disease to include the pathways of the silent mating type information regulation 2 homolog 1* (Saccharomyces cerevisiae*) (SIRT1), mammalian forkhead transcription factors (FoxOs), and AMP activated protein kinase (AMPK) (Table 1(Tab. 1)). Intimately tied to these fundamental pathways are the oversight of metabolic pathways with nicotinamide adenine dinucleotide (NAD^+^), cell senescence and lifespan, reactive oxygen species exposure, stem cell survival, mitochondrial injury, trophic factor protection, infectious agent injury, and programmed cell death pathways of apoptosis, autophagy, and ferroptosis.

## Cardiovascular Disease, SIRT1, and Cellular Metabolism

Silent mating type information regulation 2 homolog 1* (Saccharomyces cerevisiae*) (SIRT1) plays a significant role during the onset and progression of cardiovascular disease (Charles et al., 2017;[31] Cui et al., 2017[55]; Kostić et al., 2023[124]; Maiese, 2016[190], 2017[171], 2020[172][182], 2021[181][192]; Ministrini et al., 2021[225]; Piao et al., 2021[249]; Watroba and Szukiewicz, 2021[312]; Yuan et al., 2022[331]) (Table 1(Tab. 1)). A member of the sirtuin family (sirtuin 1), SIRT1 is a histone deacetylase that oversees DNA transcription by transferring acetyl groups from ε-N-acetyl lysine amino acids to the histones of DNA (Chong et al., 2012[50]; Ding et al., 2022[59]; Guimera et al., 2022[88]; Jahan et al., 2023[109]; Jalgaonkar et al., 2022[110]; Kostić et al., 2023[124]; Liu et al., 2023[153]; Maiese, 2017[171], 2020[161], 2021[197]; Sadria et al., 2022[267]; Sun et al., 2023[296]). The coenzyme ß-nicotinamide adenine dinucleotide (NAD^+^) is used as a substrate for SIRT1 (Chong et al., 2005[44], 2022[40]; Fangma et al., 2022[68]; Giacalone et al., 2021[82]; Jobst et al., 2023[115]; Maiese, 2015[191], 2016[198], 2020[179], 2021[181]; Maiese and Chong, 2003[206]; Maiese et al., 2009[210]; Ministrini et al., 2021[225]). Seven identified mammalian homologues of Sir2 include SIRT1 through SIRT7 (Cacabelos et al., 2019[22]; Maiese, 2016[196], 2018[200], Maiese, 2020[161]; Mori et al., 202[228]2; Wang et al., 2022[310]; Wasserfurth et al., 2021[311]; Zhang et al., 2020[337]). These histone deacetylases control metabolism, cell development and proliferation, senescence, and post-translation modifications of proteins (Begum et al., 2021[16]; Csicsar et al., 2019[54]; Kahmini et al., 2022[116]; Maiese, 2016[198], 2018[200], 2020[189], 2021[181]; Sun et al., 2023[296]; Tabibzadeh, 2021[298]; Wang et al., 2022[309]; Wasserfurth et al., 2021[311]; Yamamoto et al., 2023[321]; Yu et al., 2021[330]; Yuan et al., 2020[332]; Zhang et al., 2020[337]; Zhou et al., 2022[349]).

In the cardiovascular system, SIRT1 can increase the survival of cardiomyoblasts (Passariello et al., 2011[245]), lead to enhanced endothelial function (Charles et al., 2017[31]; Hajibabaie et al., 2022[92]; Maiese, 2016[187]; Piao et al., 2021[249]), maintain cardiac fatty acid oxidation (Kostić et al., 2023[124]), prevent cardiac DM injury (Maiese, 2015[191], 2017[171]; Xue et al., 2019[320]), reduce coronary artery disease (Maiese, 2020[161], 2021[177][192]; Yuan et al., 2022[331]) and block cellular senescence and impaired differentiation in endothelial progenitor cells (Lemarie et al., 2011[134]). SIRT1 can improve the function of aged stem cells that are senescent (Figure 1(Fig. 1)). Aged mesenchymal stem cells that are stimulated and pre-conditioned with glucose depletion demonstrate enhanced SIRT1 expression as well as trophic factor and protein kinase B (Akt) expression and can lead to increased cardiac performance (Choudhery et al., 2012[51]). Mesenchymal stem cells with SIRT1 over-expression exhibit increased blood vessel density in the area of cardiac infarcts, reduced cardiac remodeling, and improved cardiac performance in rodent models, suggesting SIRT1 as a potential target for the treatment of cardiac injury (Liu et al., 2014[150]).

Given that cellular metabolic dysfunction also can lead to cardiac injury and disability, the SIRT1 pathway is an important avenue in this respect since SIRT1 can oversee metabolic homeostasis (Chen et al., 2021[35]; Ghiasi et al., 2019[81]; Hajibabaie et al., 2022[92]; Hassanein et al., 2022[94]; Jalgaonkar et al., 2022[110]; Liu et al., 2021[146]; Maiese, 2015[180][188][191], 2016[183], 2020[161], 2021[181]; Wasserfurth et al., 2021[311]; Yang et al., 2020[324]). SIRT1 pathways are closely tied to NAD^+^ and the vitamin nicotinamide (Fangma et al., 2022[68]; Jobst et al., 2023[115]; Maiese, 2021[181]; Maiese et al., 2013[216]; Ministrini et al., 2021[225]; Song et al., 2019[292]; Teertam and Prakash Babu, 2021[301]; Yousafzai et al., 2021[329]; Zhang et al., 2020[337]). Nicotinamide is the amide form of the vitamin B_3 _(niacin) and a precursor for the coenzyme NAD^+^ (AlSaleh et al., 2023[6]; Fangma et al., 2022[68]; Kumar and Ou, 2023[127]; Maiese, 2020[179], 2021[181]; Rehman et al., 2022[262]; Yamamoto et al., 2023[321]; Yang et al., 2023[326]).

Nicotinamide phosphoribosyl-transferase (NAMPT) is necessary for NAD^+^ production and is controlled by SIRT1 and the circadian rhythm complex of CLOCK:BMAL1 (Maiese, 2021[181][192]). The NAMPT promoter uses SIRT1 to increase production of its own coenzyme (Nakahata et al., 2009[231]). Yet if NAD^+ ^levels are diminished, altered cellular levels of nicotinamide can result in mitochondrial dysfunction, vascular disease, and cognitive loss (Oblong, 2014[232]). This is a result of cellular NAD^+ ^pools fluctuating with circadian rhythmicity and with aging (Maiese, 2020[182]). Circadian rhythm disturbances can affect degenerative processes and metabolic dysfunction throughout the body (Amidfar et al., 2023[8]; Birnie et al., 2023[18]; Felten et al., 2023[70]; Hardeland, 2022[93]; Hsu et al., 2022[103]; Huang et al., 2023[106]; Kalam et al., 2023[117]; Klionsky et al., 2021[123]; Lathe and St Clair, 2023[130]; Luo et al., 2022[154]; Maiese, 2017[175], 2020[159][161], 2021[177]; Olejniczak et al., 2023[238]; Xu et al., 2023[319]). In relation to cellular metabolism, metformin has been reported to foster SIRT1 activity to maintain proper circadian rhythm of CLOCK and BMAL1 during obesity, since the absence of SIRT1 inhibits function of CLOCK and BMAL1 in an obese phenotype (Caton et al., 2011[25]). 

In regard to nicotinamide, SIRT1 through the transfer of the acetyl residue from the acetyllysine residue of histones to the ADP-ribose moiety of NAD^+^ can lead to the production of nicotinamide. Yet, feedback mechanisms exist and nicotinamide can block SIRT1 activity by intercepting an ADP-ribosyl-enzyme-acetyl peptide intermediate with the regeneration of NAD^+^ (Jackson et al., 2003[107]). Physiological concentrations of nicotinamide can non-competitively block SIRT1 that indicates nicotinamide is a regulator of SIRT1 (Bitterman et al., 2002[19]). As a result of SIRT1 inhibition, nicotinamide at times can suppress the expression of anti-inflammatory genes (Zhang et al., 2012[339]). However, in this process, enhanced activity of SIRT1 can occur with the activation of nicotinamide phosphoribosyltransferase (NAMPT) such as during periods of glucose restriction, resulting in increased NAD^+^ and decreased nicotinamide, an inhibitor of SIRT1 (Fulco et al., 2008[77]).

NAD^+^ replenishment is believed to foster cardiac and vascular health (Rotllan et al., 2021[266]). Enhanced NAD^+ ^levels that rely upon SIRT1 activation may reduce inflammation, metabolic instability, and cardiac injury (Maiese, 2008[203], 2015[191]; Watroba and Szukiewicz, 2021[312]) (Figure 1(Fig. 1)). Growth factors, such as erythropoietin (EPO), also may depend upon SIRT1 and NAD^+^ activity to offer cellular protection (Govindappa and Elfar, 2022[85]; Hu et al., 2022[104]; Liu et al., 2022[145]; Maiese, 2008[203], 2020[182]; Maiese et al., 2010[222]; Senousy et al., 2022[278]; Sergio and Rolando, 2022[279]). EPO can limit oxidative stress through pathways of NAD^+^ activity to preserve cellular survival in adipocytes (Wang et al., 2014[305]). EPO results in cerebral vascular protection through the subcellular trafficking of SIRT1 to the nucleus and limits mitochondrial depolarization, cytochrome c release, BCL2 associated agonist of cell death (Bad) activity, and caspase activation (Hou et al., 2011[102]). Through SIRT1 activation, EPO can increase survival of human cardiomyocytes (Cui et al., 2017[55]), control metabolic pathways (Entezari et al., 2019[66]; Fessel, 2023[73]; Montesano et al., 2019[227]; Yang et al., 2023[325]), prevent mitochondrial dysfunction (Chong et al., 2002[43], 2003[42]; Cui et al., 2017[55]; Maiese, 2016[190]; Rey et al., 2021[264]; Shang et al., 2012[284]), foster microglial survival (Shang et al., 2011[283]), and inhibit caspase activity that leads to apoptotic cell death (Shang et al., 2012[285]). 

## Cardiovascular Disease and Mammalian Forkhead Transcription Factors (FoxOs)

Mammalian forkhead transcription factors (FoxOs) can affect the cardiovascular system through a number of pathways that involve oxidative stress, programmed cell death, and metabolic homeostasis (Abuzenadah et al., 2018[2]; Du et al., 2016[64]; Kandula et al., 2016[118]; Klionsky et al., 2021[123]; Kostić et al., 2023[124]; Maiese et al., 2009[209][214][218], 2012[217]; Razzaghi et al., 2023[260]; Schips et al., 2011[277]) (Table 1(Tab. 1)). During oxidative stress and programmed cell death, inhibition of FoxO activity can prevent microglial cell apoptosis during oxidative stress (Czubowicz et al., 2019[56]; Guo et al., 2017[89]; Hong et al., 2012[100]; Shang et al., 2009[281]; Shi et al., 2016[288]), promote cellular protection through metabotropic glutamate receptors (Maiese, 2015[169]), and block apoptotic cell death through NAD^+^ precursors (AlSaleh et al., 2023[6]; Chong et al., 2022[40]; Kumar and Ou, 2023[127]; Lin et al., 2022[143]; Maiese, 2008[203], 2020[179], 2021[181]; Maiese and Chong, 2003[206]; Maiese et al., 2009[210]; Rehman et al., 2022[262]; Wang et al., 2022[309]; Ye et al., 2022[327]) (Figure 1(Fig. 1)). Metformin can prevent apoptotic cell death in ischemic myocardium through the down-regulation of FoxO3a and the inhibition of caspase activation (Elmadhun et al., 2014[65]). Once FoxO proteins become active, cytochrome c release can occur with caspase-induced apoptotic death (Hou et al., 2010[101]; Qi et al., 2013[254]; Shang et al., 2010[282]; Shi et al., 2016[288]). Similar pathways apply with inhibition of FoxO activity to preserve cardiac cell survival during DM cardiomyopathy (Kandula et al., 2016[118]; Maiese et al., 2009[218]) and cardiac ischemic-perfusion injury (Guan et al., 2016[87]; Qi et al., 2013[254]). In relation to metabolic pathways with nicotinamide, phosphorylation of FoxO3a by nicotinamide at regulatory sites that possess high affinity for Akt can block apoptotic cell injury (Chong et al., 2004[46]). Nicotinamide offers cellular protection through two mechanisms of post-translational modification of FoxO3a (Maiese et al., 2008[211], 2009[214][213]). One mechanism maintains phosphorylation of FoxO3a and inhibits caspase 3 activity (Chong et al., 2004[46]). With the second mechanism, nicotinamide can preserve the integrity of the FoxO3a protein to block FoxO3a proteolysis. If FoxO3a does not become fragmented, then the generation of “pro-apoptotic” amino-terminal (Nt) fragments that normally would result is prevented (Charvet et al., 2003[32]). Similar to the metabolic pathways of nicotinamide, EPO is dependent upon FoxOs to prevent apoptotic cell loss (Fessel, 2023[73][74]; Govindappa and Elfar, 2022[85]; Hu et al., 2022[104]; Maiese, 2016[190], 2023[158]; Maiese et al., 2005[220]; Senousy et al., 2022[278]; Sergio and Rolando, 2022[279]; Yang et al., 2023[325]). EPO leads to post-translational phosphorylation of FoxO3a (Chong et al., 2011[41]), fosters FoxO3a and 14-3-3 protein binding, and oversees the intracellular trafficking of FoxO3a (Chong and Maiese, 2007[47]; Hou et al., 2011[102]). EPO is able to reverse the acetylation of FOXO3a and FOXO1a (Mahmud et al., 2002[156]) and decrease transcriptional activity of FoxO1 (Maiese, 2021[197]; Maiese et al., 2009[209]; Zhao et al., 2015[343]).

Although FoxO proteins are expressed throughout the body, FoxOs maintain a significant role in the cardiovascular system (Abuzenadah et al., 2018[2]; Blice-Baum et al., 2017[20]; Du et al., 2016[64]; Kostić et al., 2023[124]; Maiese, 2020[165], 2021[160], 2023[158]; Margrett et al., 2022[223]). Mammalian FOXO proteins of the O class include the members FOXO1, FOXO3, FOXO4, and FOXO6 (Jalgaonkar et al., 2022[110]; Ji, Liu et al., 2022[113]; Maiese et al., 2008[211], 2009[213]; Salcher et al., 2020[269]; Salih et al., 2012[271]). The function of FoxO proteins is conserved among multiple species that include *Caenorhabditis elegans, Drosophila melanogaster,* and mammals. FoxO proteins are homologous to the transcription factor DAuer Formation-16 (DAF-16) in the worm *Caenorhabditis elegans* that can oversee metabolic insulin signaling, cell survival, cell cycle regulation, and lifespan extension (Lin et al., 1997[142]; Ogg et al., 1997[236]; Sangaletti et al., 2017[273]). FoxO proteins also affect related systems to the cardiac system that involve cerebral endothelial vascular cell survival (Hou et al., 2011[102]; Maiese et al., 2004[219]), cerebral traumatic injury (Liu et al., 2021[151]), and gluconeogenesis (Calabuig-Navarro et al., 2015[24]). FoxOs are controlled by epigenetic and post-translational protein modifications that involve phosphorylation (Maiese, 2015[169]; Peng et al., 2020[247]; Zeng et al., 2020[336]), ubiquitylation (Zeldich et al., 2014[335]), and acetylation (BinMowyna and AlFaris, 2021[17]; Ren et al., 2021[263]; Shati and El-Kott, 2021[287]). Forkhead transcription factor phosphorylation is modulated by Akt (Maiese, 2016[190], 2020[189]) such that Akt phosphorylates FoxO proteins to promote binding to 14-3-3 proteins, block nuclear translocation, and inhibit transcription of target genes that would ultimately lead to apoptosis (Maiese, 2015[169]; Sanphui et al., 2020[274]; Wang et al., 2013[307]). Akt also leads to the ubiquitination and degradation of FoxOs through the 26S proteasome. FoxOs are acetylated by histone acetyltransferases that include the CREB-binding protein (CBP), the CBP-associated factor, and p300. After FoxOs undergo acetylation, nuclear translocation of FoxOs ensues but FoxO proteins now have diminished activity. This loss of FoxO activity occurs as a result of the acetylation of lysine residues on FoxO proteins limiting the ability of FoxO proteins to bind to DNA (BinMowyna and AlFaris, 2021[17]; Farhan et al., 2017[69]; Ren et al., 2021[263]; Shati and El-Kott, 2021[287]). Interestingly, acetylation of FoxOs results in the phosphorylation of FoxOs by Akt (Matsuzaki et al., 2005[224]). 

In a number of scenarios, the inhibition of FoxO activity can be beneficial for cell survival and the prevention of apoptotic pathways (BinMowyna and AlFaris, 2021[17]; Cheema et al., 2021[33]; Farhan et al., 2017[69]; Gökdoğan Edgünlü et al., 2020[83]; He et al., 2021[97]; Liu et al., 2020[149]; Maiese, 2015[169][170], 2016[168]; Sanphui et al., 2020[274]; Sharma et al., 2021[286]; Shati and El-Kott, 2021[287]; Zeng et al., 2020[336]; Zhao et al., 2023[341][346]). Yet, FoxOs can enhance survival during the activation of autophagy mediated pathways (Maiese, 2016[168], 2018[184], 2021[177]). With FoxO1 activation and the induction of autophagy, basal autophagy is increased that can reduce atherogenesis (Maiese, 2015[169]; Weikel et al., 2016[313]). Exercise induced activation of autophagy results in the down-regulation of FoxO3a and suppression of sarcopenia (Zeng et al., 2020[336]). Autophagy activation in association with modulation of FoxO signaling also results in decreased renal tubulointerstitial fibrosis (Zhao et al., 2021[344]), protection through metformin to reduce inflammation (Ali et al., 2020[5]), and reduction of cardiotoxicity during ferroptosis (He et al., 2021[97]). A limited degree of FoxO activation may be required to promote cellular survival with autophagy during cardiac injury. FoxOs through the activation of autophagy can lead to the clearance of toxic intracellular accumulations and promote neuronal survival (Saleem and Biswas, 2017[270]; Tabibzadeh, 2021[298]). 

In addition to pathways of programmed cell death, oxidative stress, and cellular metabolism, FoxOs share a close relationship with SIRT1. SIRT1 can be involved with DNA transcription by transferring acetyl groups from ε-N-acetyl lysine amino acids to the histones of DNA. As a result, FoxO acetylation can be controlled by SIRT1 and other histone deacetylases (Kostić et al., 2023[124]; Li et al., 2020[139]; Maiese, 2018[186], 2021[177]; Rong et al., 2021[265]; Shati and El-Kott, 2021[287]; Yaman et al., 2020[322]). Increased SIRT1 activity can modify FoxO activity to reduce oxidative stress during cardiac ischemia (Guan et al., 2016[87]), protect against vascular cerebral injury (Hassanein et al., 2022[94]), limit DM complications (Jalgaonkar et al., 2022[110]), repair microcirculation disturbances (Rong et al., 2021[265]), improve cardiac left ventricular remodeling during renal disease (Li et al., 2020[139]), activate senescence mesenchymal stem cells through telomerase activity (Okada et al., 2016[237]), and assist with cardiac fatty acid metabolism (Kostić et al., 2023[124]). In addition, there exists an autofeedback mechanism to regulate SIRT1 activity through FoxOs. FoxOs can bind to the SIRT1 promoter region that contains a cluster of five putative core binding repeat motifs (IRS-1) and a forkhead-like consensus-binding site (FKHD-L) to modify the transcription of forkhead. FoxO proteins can oversee SIRT1 transcription and increase SIRT1 expression (Xiong et al., 2011[318]). FoxOs and SIRT1 also can function in a synergistic manner to increase cell survival and have been shown to prevent mitochondrial dysfunction during oxidative stress (Lin et al., 2015[141]).

## Cardiovascular Disease and AMP-activated protein kinase (AMPK)

AMP-activated protein kinase (AMPK), a member of the mechanistic target of rapamycin (mTOR) pathway (Chong and Maiese, 2012[48]; Chong et al., 2011[49]; Hua et al., 2023[105]; Maiese, 2014[164][195], 2016[196], 2020[161][165]; Thomas et al., 2023[303]; Zhang et al., 2023[340]; Zhao et al., 2023[347]), is vital in the control of cellular metabolism and insulin sensitivity that can impact cardiovascular disease (Barcena et al., 2023[13]; Castano et al., 2014[27]; Dong et al., 2019[60]; Hua et al., 2023[105]; Maiese, 2016[196], 2017[171], 2020[165], 2023[158]; Pal et al., 2019[243]; Watroba and Szukiewicz, 2021[312]; Yang et al., 2020[324]; Zhong et al., 2023[348]) (Table 1(Tab. 1)). AMPK and mTOR also may employ metabolic pathways during the control of severe acute respiratory syndrome coronavirus (SARS-CoV-2) and coronavirus disease 2019 (COVID-19) (Abu-Eid and Ward, 2021[1]; Alves et al., 2022[7]; Bramante et al., 2023[21]; Khan, 2021[119]; Khan et al., 2021[120]; Liu et al., 2023[153]; Maiese, 2020[201][182], 2021[202], 2022[157], 2023[158]; Pinchera et al., 2022[250]; Swain et al., 2021[297]) (Figure 1(Fig. 1)). Diets with fish oil consumption increase AMPK activity and prevent endothelial progenitor cell dysfunction (Chiu et al., 2017[39]). AMPK limits insulin resistance (Liu et al., 2014[152]) and may increase lifespan (Balan et al., 2008[11]) since AMPK can be one of multiple pathways to shift to beneficial oxidative metabolism (Moroz et al., 2014[229]). Nicotinamide may limit mitochondrial stress through AMPK activation (Lai et al., 2019[128]). Metformin also has a role with AMPK. Biguanides and metformin rely upon AMPK and autophagy to maintain cellular function. Through metformin, AMPK is activated, results in autophagy induction, and protects against DM apoptotic cardiac cell loss (He et al., 2013[96]). Metformin limits lipid peroxidation in the brain and spinal cord and decreases caspase activity during toxic insults (Oda, 2017[233]). These observations of metformin to offer protection during metabolic dysfunction may be associated with the ability of autophagic pathways to limit oxidative stress (Amidfar et al., 2023[8]; Chong et al., 2005[45]; Ciesielska and Gajewska, 2023[52]; du Toit et al., 2023[62]; Maiese, 2017[204], 2018[200], 2020[189], 2023[158]; Raghuvanshi et al., 2023[256]; Raut and Khullar, 2023[259]; Zhong et al., 2023[348]). Under conditions of metabolic dysfunction, AMPK can control programmed cell death during coronary artery disease (Dong et al., 2019[60]), cholesterol efflux (An et al., 2020[9]), endothelial dysfunction during hyperglycemia (Pal et al., 2019[243]), oxidative stress (Shokri Afra et al., 2019[289]; Zhao et al., 2019[342]), and prevent mitochondrial dysfunction during ferroptosis (Zhong et al., 2023[348]). In the absence of AMPK function, cell injury, cell senescence, and mitochondrial dysfunction can ensue and lead to cardiomyopathy (Barcena et al., 2023[13]; Watroba and Szukiewicz, 2021[312]). AMPK activity limits myocardial infarct size in both non-diabetic and diabetic rat hearts following exposure to ischemia/reperfusion. This process may be controlled through the inhibition of mitochondrial permeability transition pore opening in cardiomyocytes (Paiva et al., 2011[242]). 

SIRT1 is a principal pathway in overseeing the activity of AMPK that can be affected by aging, inflammation, and tumorigenesis (Guimera et al., 2022[88]; Maiese, 2016[167], 2020[182]; Sadria et al., 2022[267]; Yang et al., 2020[324]; Yu et al., 2021[330]). SIRT1 functions through the AMPK kinase, serine-threonine liver kinase B1 (LKB1). Over-expression of SIRT1 can lead to the deacetylation of LKB1 and produce the translocation of LKB1 from the nucleus to the cytoplasm to activate AMPK (Lan et al., 2008[129]). As a result, AMPK enhances SIRT1 activity but it is believed not to be through a direct mechanism. AMPK activation increases SIRT1 activity either by increasing cellular NAD^+^/NADH ratio, resulting in the deacetylation and modulation of the activity of downstream SIRT1 targets that include peroxisome proliferator-activated receptor-gamma coactivator-1α (PGC-1α), FoxO1, and FoxO3a (Canto and Auwerx, 2009[25]) or by up-regulating NAMPT during glucose restriction, leading to increased NAD^+^ and decreased activity of nicotinamide that can inhibit SIRT1 (Fulco et al., 2008[77]). The SIRT1 activator resveratrol can increase AMPK activity through SIRT1 dependent or independent mechanisms (Canto and Auwerx, 2009[25]; Herranz and Serrano, 2010[99]). 

## Future Perspectives

Throughout the world, cardiovascular disease is the leading cause of death for both women and men to the extent that an individual expires every thirty-three seconds as a result of heart disease in just the US alone. Healthcare costs for cardiovascular disease can be greater than $ 555 billion US dollars and are expected to exceed $ 1.1 trillion US dollars by the year 2025. Cardiovascular disease is affected by the increased lifespan of the global population that also leads to cellular pathways that mediate progressive cellular oxidative stress, the shortening of TLs, and the inception of cell senescence. The onset of cardiovascular disease has additional risks that involve age sixty and over, lower income, high serum cholesterol levels, elevated blood pressure, tobacco consumption and secondhand smoke exposure, poor nutrition, physical inactivity, obesity, and the existence of DM. Although treatment of disorders such as DM can lessen the risk of developing cardiovascular disease, cardiac disease may continue to progress and the risk of additional complications may develop, such as the loss of organ mass. These considerations for the development of novel and effective treatments for cardiovascular disease call for innovative strategies that involve SIRT1, FoxOs, and AMPK.

SIRT1, a histone deacetylase that can impact DNA transcription, can oversee metabolic homeostasis through NAD^+^ pathways and increase cardiomyoblast survival, improve stem cell development, assist with endothelial cell function, reduce coronary artery disease, limit cell senescence, and prevent cardiac injury during DM. SIRT1 also promotes necessary circadian rhythm pathways during obesity and treatment with metformin, mediates growth factor protection with EPO, and leads to the production of nicotinamide to maintain mitochondrial function. Yet, feedback pathways are necessary between SIRT1 and nicotinamide to effectively foster anti-inflammatory pathways. 

Pathways of SIRT1 are also closely tied to FoxO activity. SIRT1 activation can oversee FoxO cardiac activity to limit oxidative stress, reduce DM complications, repair microcirculation disturbances, improve left ventricular remodeling, and activate senescence mesenchymal stem cells through telomerase activity. Feedback pathways exist for SIRT1 and FoxOs such that forkhead transcription can be altered and that FoxOs can increase SIRT1 expression. This intimate relationship is vital since under some scenarios, the increased activity of FoxOs rather than inhibition of FoxO activity can promote cell survival during cardiotoxicity and ferroptosis through the activation of autophagy pathways. With other circumstances, FoxOs and SIRT1 can function in a synergistic manner to increase cell survival and block mitochondrial dysfunction during oxidative stress. 

The pathways of SIRT1 and FoxOs are also complemented by the mTOR pathway AMPK. AMPK through autophagy activation can modulate infections with COVID-19, reduce endothelial dysfunction, increase lifespan, improve insulin sensitivity, and assist nicotinamide to maintain mitochondrial function. Furthermore, AMPK can function through the application of metformin to block cardiac apoptosis during DM, reduce cardiac infarct size, and limit cellular senescence. SIRT1 also is vital in the AMPK pathway to not only oversee AMPK activity, but also for AMPK to increase SIRT1 activation to limit cardiomyopathy, cell injury, and mitochondrial dysfunction.

The cellular pathways of SIRT1, FoxOs, and AMPK offer highly novel considerations to address the onset and development of cardiovascular disease. These pathways are significant in the ability to modulate cellular oxidative stress, metabolic pathways with NAD^+^, cell senescence and lifespan, mitochondrial injury, trophic factor protection, infectious agent injury, and programmed cell death pathways that include apoptosis, autophagy, and ferroptosis. However, the relationship among these pathways is highly complex with the existence of multiple feedback systems that will require a continued focus on new insights to safely and effectively target these innovative mechanisms for current and future clinical translation in the treatment of cardiovascular disease.

## Declaration

### Acknowledgments

This research was supported by the following grants to Kenneth Maiese: American Diabetes Association, American Heart Association, NIH NIEHS, NIH NIA, NIH NINDS, and NIH ARRA.

### Conflict of interest

The author declares no conflict of interest.

## Figures and Tables

**Table 1:**
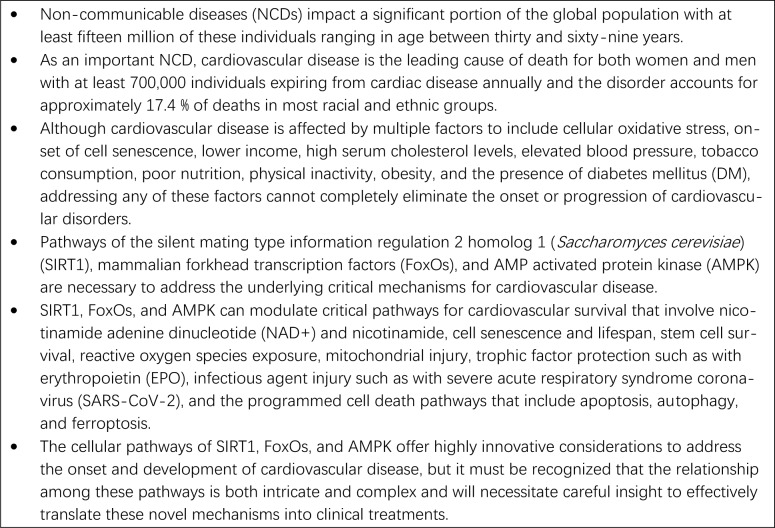
Highlights "Novel therapeutic strategies for cardiovascular disease"
